# Supplementary material for: Modeling glioblastoma heterogeneity as a dynamic network of cell states
Source: Mol Syst Biol. 2021 Sep 16;17(9):e10105. doi: 10.15252/msb.202010105 (PMC8444284; doi:10.15252/msb.202010105)
Supplement: Supplementary file 6 — Source Data for Figure 5 [file MSB-17-e10105-s004.zip › Figure5A_sourcedata/GSEA_3071/hallmarks_stateI.GseaPreranked.1623742531165/HALLMARK_UV_RESPONSE_UP.html]

Details for gene set HALLMARK\_UV\_RESPONSE\_UP[GSEA]

|  || Dataset | state13071 |
| Phenotype | NoPhenotypeAvailable |
| Upregulated in class | na\_neg |
| GeneSet | HALLMARK\_UV\_RESPONSE\_UP |
| Enrichment Score (ES) | -0.21427615 |
| Normalized Enrichment Score (NES) | -0.99904627 |
| Nominal p-value | 0.47678018 |
| FDR q-value | 0.61986446 |
| FWER p-Value | 1.0 |
Table: GSEA Results Summary

  

Fig 1: Enrichment plot: HALLMARK\_UV\_RESPONSE\_UP      
 Profile of the Running ES Score & Positions of GeneSet Members on the Rank Ordered List

  

| PROBE | GENE SYMBOL | GENE\_TITLE | RANK IN GENE LIST | RANK METRIC SCORE | RUNNING ES | CORE ENRICHMENT || 1 | MMP14 |  |  | 27 | 1.526 | 0.0414 | No |
| 2 | CDKN2B |  |  | 112 | 1.047 | 0.0508 | No |
| 3 | IRF1 |  |  | 276 | 0.705 | 0.0263 | No |
| 4 | SQSTM1 |  |  | 412 | 0.578 | 0.0058 | No |
| 5 | EPHX1 |  |  | 429 | 0.570 | 0.0195 | No |
| 6 | JUNB |  |  | 454 | 0.546 | 0.0302 | No |
| 7 | PDLIM3 |  |  | 547 | 0.481 | 0.0190 | No |
| 8 | RHOB |  |  | 609 | 0.447 | 0.0157 | No |
| 9 | HMOX1 |  |  | 623 | 0.443 | 0.0262 | No |
| 10 | FURIN |  |  | 663 | 0.426 | 0.0287 | No |
| 11 | TUBA4A |  |  | 664 | 0.425 | 0.0424 | No |
| 12 | FOS |  |  | 832 | 0.370 | 0.0059 | No |
| 13 | FOSB |  |  | 854 | 0.364 | 0.0116 | No |
| 14 | TYRO3 |  |  | 895 | 0.352 | 0.0113 | No |
| 15 | PPT1 |  |  | 1071 | 0.312 | -0.0293 | No |
| 16 | IL6ST |  |  | 1215 | 0.285 | -0.0615 | No |
| 17 | TMBIM6 |  |  | 1391 | 0.253 | -0.1040 | No |
| 18 | STIP1 |  |  | 1453 | -0.253 | -0.1135 | No |
| 19 | CCND3 |  |  | 1482 | -0.255 | -0.1134 | No |
| 20 | CDKN1C |  |  | 1571 | -0.264 | -0.1304 | No |
| 21 | CNP |  |  | 1582 | -0.265 | -0.1248 | No |
| 22 | CASP3 |  |  | 1639 | -0.273 | -0.1322 | No |
| 23 | PPAT |  |  | 1655 | -0.275 | -0.1277 | No |
| 24 | WIZ |  |  | 1704 | -0.280 | -0.1326 | No |
| 25 | ENO2 |  |  | 1707 | -0.281 | -0.1241 | No |
| 26 | NUP58 |  |  | 1711 | -0.281 | -0.1159 | No |
| 27 | PSMC3 |  |  | 1752 | -0.287 | -0.1183 | No |
| 28 | TST |  |  | 1779 | -0.291 | -0.1164 | No |
| 29 | ACAA1 |  |  | 1862 | -0.300 | -0.1305 | No |
| 30 | KCNH2 |  |  | 1937 | -0.311 | -0.1419 | No |
| 31 | PPP1R2 |  |  | 2065 | -0.328 | -0.1681 | No |
| 32 | E2F5 |  |  | 2082 | -0.331 | -0.1621 | No |
| 33 | CCNE1 |  |  | 2087 | -0.332 | -0.1525 | No |
| 34 | MRPL23 |  |  | 2130 | -0.339 | -0.1538 | No |
| 35 | ARRB2 |  |  | 2235 | -0.360 | -0.1723 | No |
| 36 | BTG2 |  |  | 2318 | -0.375 | -0.1839 | No |
| 37 | BID |  |  | 2405 | -0.393 | -0.1962 | No |
| 38 | HNRNPU |  |  | 2434 | -0.398 | -0.1915 | No |
| 39 | HYAL2 |  |  | 2483 | -0.408 | -0.1922 | No |
| 40 | PARP2 |  |  | 2542 | -0.425 | -0.1953 | No |
| 41 | POLE3 |  |  | 2570 | -0.432 | -0.1892 | No |
| 42 | PDAP1 |  |  | 2581 | -0.435 | -0.1781 | No |
| 43 | BMP2 |  |  | 2589 | -0.436 | -0.1661 | No |
| 44 | PPIF |  |  | 2624 | -0.446 | -0.1616 | No |
| 45 | CDC34 |  |  | 2769 | -0.495 | -0.1873 | No |
| 46 | CDK2 |  |  | 2863 | -0.526 | -0.1973 | Yes |
| 47 | FKBP4 |  |  | 2871 | -0.528 | -0.1824 | Yes |
| 48 | CYP1A1 |  |  | 2886 | -0.533 | -0.1693 | Yes |
| 49 | GGH |  |  | 2975 | -0.585 | -0.1759 | Yes |
| 50 | OLFM1 |  |  | 2981 | -0.589 | -0.1583 | Yes |
| 51 | GAL |  |  | 3046 | -0.631 | -0.1566 | Yes |
| 52 | NPTXR |  |  | 3114 | -0.687 | -0.1538 | Yes |
| 53 | RFC4 |  |  | 3129 | -0.701 | -0.1353 | Yes |
| 54 | BTG3 |  |  | 3148 | -0.727 | -0.1171 | Yes |
| 55 | AGO2 |  |  | 3153 | -0.732 | -0.0947 | Yes |
| 56 | SELENOW |  |  | 3209 | -0.795 | -0.0850 | Yes |
| 57 | FEN1 |  |  | 3278 | -0.890 | -0.0760 | Yes |
| 58 | SLC25A4 |  |  | 3316 | -0.953 | -0.0560 | Yes |
| 59 | IGFBP2 |  |  | 3509 | -3.484 | 0.0006 | Yes |
Table: GSEA details [plain text format]

  

Fig 2: HALLMARK\_UV\_RESPONSE\_UP: Random ES distribution      
 Gene set null distribution of ES for **HALLMARK\_UV\_RESPONSE\_UP**
